# Marine Heterocyclic Compounds That Modulate Intracellular Calcium Signals: Chemistry and Synthesis Approaches

**DOI:** 10.3390/md19020078

**Published:** 2021-01-31

**Authors:** Paula González-Andrés, Laura Fernández-Peña, Carlos Díez-Poza, Carlos Villalobos, Lucía Nuñez, Asunción Barbero

**Affiliations:** 1 Department of Organic Chemistry, Campus Miguel Delibes, University of Valladolid, 47011 Valladolid, Spain; paula.gonzalez.andres@alumnos.uva.es (P.G.-A.); laura.fernandez.pena@uva.es (L.F.-P.); carlos.diez@uva.es (C.D.-P.); 2 Institute of Biology and Molecular Genetics (IBGM), University of Valladolid and Spanish National Research Council (CSIC), 47003 Valladolid, Spain; carlosv@ibgm.uva.es (C.V.); nunezl@ibgm.uva.es (L.N.); 3 Department of Biochemistry and Molecular Biology and Physiology, University of Valladolid, 47005 Valladolid, Spain

**Keywords:** marine drugs, heterocycles, calcium channel, total synthesis, medicinal properties

## Abstract

Intracellular Ca^2+^ plays a pivotal role in the control of a large series of cell functions in all types of cells, from neurotransmitter release and muscle contraction to gene expression, cell proliferation and cell death. Ca^2+^ is transported through specific channels and transporters in the plasma membrane and subcellular organelles such as the endoplasmic reticulum and mitochondria. Therefore, dysregulation of intracellular Ca^2+^ homeostasis may lead to cell dysfunction and disease. Accordingly, chemical compounds from natural origin and/or synthesis targeting directly or indirectly these channels and proteins may be of interest for the treatment of cell dysfunction and disease. In this review, we show an overview of a group of marine drugs that, from the structural point of view, contain one or various heterocyclic units in their core structure, and from the biological side, they have a direct influence on the transport of calcium in the cell. The marine compounds covered in this review are divided into three groups, which correspond with their direct biological activity, such as compounds with a direct influence in the calcium channel, compounds with a direct effect on the cytoskeleton and drugs with an effect on cancer cell proliferation. For each target, we describe its bioactive properties and synthetic approaches. The wide variety of chemical structures compiled in this review and their significant medical properties may attract the attention of many different researchers.

## 1. Introduction

A variety of heterocyclic compounds modulate different biological functions. Here we will focus on heterocyclic compounds that affect intracellular calcium signal through their effect on ion channels, cytoskeleton and impact on tumor cell proliferation.

The divalent cation Ca^2+^ is a fundamental ion in cellular metabolism as well as participating in the construction and maintenance of bones and teeth. Furthermore, ionic intracellular Ca^2+^ is also critically involved in cell signaling, as this ion is considered one of the most important second messengers. Thus, intracellular Ca^2+^ ions modulate a large number of cell functions from exocytosis and muscle contraction to regulation of gene expression, cell proliferation and cell death [1,2]. Intracellular Ca^2+^ is also a particular second messenger because it is neither created nor destroyed inside the cell; instead, it is transported throughout the cell and intracellular membranes by means of channels and other specific transport systems that play an essential role by regulating its intracellular concentration. Ca^2+^ binds and modifies the activity of hundreds of Ca^2+^-binding proteins like, for instance, calmodulin that, in turn, modify the activity of another even larger series of critical enzymes and other proteins [3]. This is how it is involved in regulating muscle contraction, hormone secretion and neurotransmitter exocytosis, gene expression, cell mobility, apoptosis, fertilization or synaptic plasticity, among other functions [4,5]. It is also involved in the regulation of some kinases, such as protein kinase C, as the action of cofactor or other enzymes of the Krebs cycle, which entails regulation of cellular energy metabolism [6]. All of these processes occur as a result of variations in the cytosolic Ca^2+^ concentration that are characterized by its amplitude, duration, frequency and subcellular location [7]. The transport of Ca^2+^ in the cell is possible by an enormous electrochemical gradient. At rest, cytosolic Ca^2+^ is 50–100 nM, while outside of the cell, the concentration is between 1 and 2 mM. Within the cell, the main storage for Ca^2+^ is the endoplasmic reticulum with concentrations in the 1–2 mM level at rest. However, in other subcellular organelles such as mitochondria and the nucleus, the resting [Ca^2+^] is similar to the cytosol and can only be increased temporarily after stimulation [8]. Electrical gradients such as plasma membrane potential, negative inside the inner part of the plasma membrane, and the huge mitochondrial potential, also negative inside the mitochondrial matrix, play a pivotal role. They favor Ca^2+^ entry from outside to the cytosol as well as mitochondrial Ca^2+^ uptake from the cytosol, in this latter case, even in the absence of Ca^2+^ chemical gradients. However, in contrast to the endoplasmic reticulum (ER), mitochondria are not Ca^2+^ stores, they may take Ca^2+^ quickly, particularly from high Ca^2+^ concentration hotspots, but this Ca^2+^ returns slowly back to the cytosol [9].

Ion channels are transmembrane proteins involved in the passive transport of ions, both cations and anions, across biological membranes as the plasma membrane of eukaryotic cells or endomembranes that limit subcellular organelles such as the endoplasmic reticulum, the nuclear envelope or mitochondria, to cite the most important ones. Ion channels are important to cells because they modulate electric gradients required for life. In addition, ion channels that transport the divalent cation Ca^2+^ are critically involved in cell signaling. Therefore, many drugs from natural origin and/or chemical synthesis, including several marine heterocyclic compounds that target calcium signaling, are potentially useful for preventing and/or treating multiple diseases, including some of the most prevalent ones from hypertension to cancer and Alzheimer’s disease [10].

The cytoskeleton is a subcellular structure made of different proteins and other biological compounds that subserve important cell functions across cell lifespan from cell division to cell differentiation, cell migration and cell death. Specifically, cytoskeleton actin filaments play an important role in the modulation of ion channel function, including Ca^2+^ channels that are a key for exocytosis; as a consequence, actin filaments are essential in exocytosis [11]. Accordingly, chemical compounds targeting any of the proteins involved in the cytoskeleton may have a deep impact on the pathophysiology of the different cell types.

One of the most critical threats to human life is cancer. Cancer is, in most cases, the result of cell transformation secondary to mutations either inherited, spontaneous, /or induced by physical or chemical factors in genes involved in critical pathways of cell signaling related to cell proliferation or DNA repair. The number of signaling pathways and critical regulatory proteins is large but still limited, and changes in the activity of any of these proteins may have an impact on cell proliferation and cancer development. Ca^2+^ signaling contributes direct or indirectly to many of these tumor characteristics. Multiple studies suggest changes in the intracellular Ca^2+^ homeostasis across carcinogenesis; thus, Ca^2+^ entry is necessary for all tumor cell proliferation [12,13]. Hundreds of chemical compounds, including some heterocycles, have an impact on calcium-dependent cell proliferation and therefore have been considered as a drug for cancer treatment.

**Figure 1:**
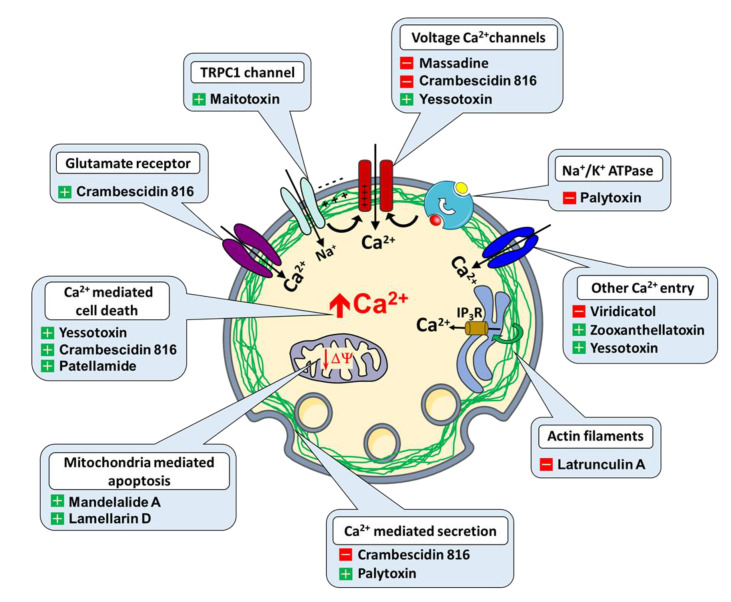
Ion channels, transport proteins and Ca^2+^ regulated functions targeted by heterocycles (+ activator, − inhibitor).

In this review, we have tried to cover marine drugs containing heterocycle subunits, which have a direct influence on intracellular calcium signal (Figure 1). Our aim is to provide a general overview of their biological properties, effects on the calcium channels and synthetic approaches to these compounds. The review is organized into three main sections, which are related to their principal biological properties: effects over calcium channels, effects over cytoskeleton and effects on tumor cell proliferation.

## 2. Heterocyclic Marine Drugs with Effect over Calcium Channels

### 2.1. Viridicatol

**Figure 2:**
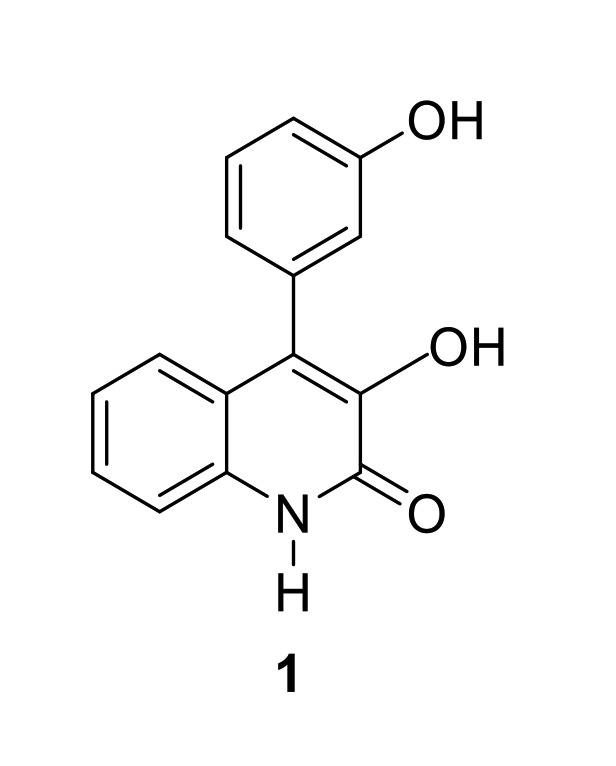
Viridicatol.

3-hydroxy-4-(3-hydroxyphenyl)-1H-quinolin-2-one, also named viridicatol, is a quinoline alkaloid obtained from a deep-sea derived fungus of the genus *Penicillium griseofulvum* (Figure 2).

Recent data suggest that, in mast cells, viridicatol may inhibit Ca^2+^ signaling induced by ionophores. As a consequence, viridicatol may prevent mast cell degranulation, which could block the activation of mast cells that are involved in the inflammatory response [14]. Evidence showed that viridicatol also abolishes intracellular Ca^2+^ increases in rat basophilic leukemia cells and mast cells isolated from mice treated with ovalbumin to induce food allergy, thus alleviating allergic symptoms. Accordingly, this compound may be considered to treat and/or prevent allergic reactions mediated by mast cell activation and degranulation.

**Scheme 1:**
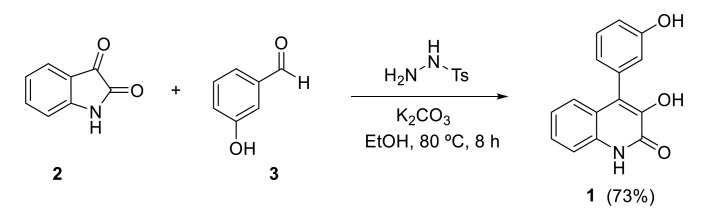
Synthesis of viridicatol by Kamal and Babu.

Regarding its total synthesis, in 1964, Luckner and Mohammed [15] described an early approach to viridicatol **1** based on an unexpected regioselective ring expansion process in the reaction of isatin with α-aryldiazomethane (prepared by reaction of an aryl aldehyde with hydrazine and subsequent oxidation of the arylhydrazone intermediate with mercuric oxide). Later on, Kamal, Babu and coworkers reported an improved one-pot methodology for the synthesis of viridicatol [16]. In this protocol, *N*-tosylhydrazine adds to aldehyde **3** in the presence of a base (K_2_CO_3_) to generate in situ the α-aryldiazomethane, which then reacts with isatin **2** to obtain quinolinone viridicatol **1** (Scheme 1).

The discovery of the promising biological properties of this marine drug, reported in the late nineties, inspired the development of new synthetic approaches.

**Scheme 2:**
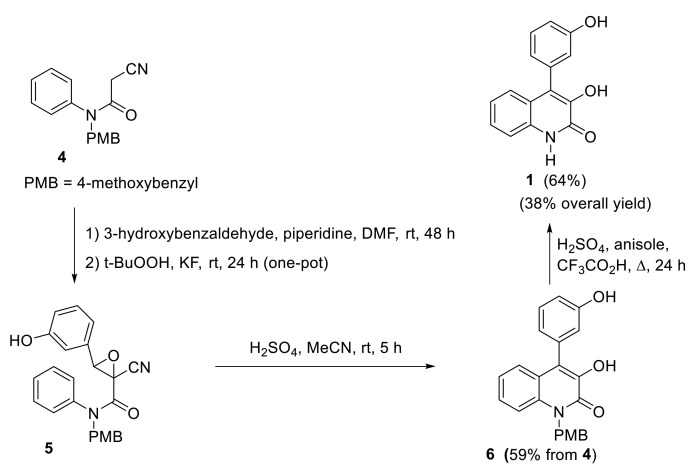
Kobayashi and Harayama’s synthesis of viridicatol.

Kobayashi and Harayama reported a synthetic approach for viridicatol based on a Knoevenagel condensation of cyanoacetanilides with aldehydes, followed by an oxidative cyclization, in which the cyano substituent works as a leaving group [17]. Cyanoacetanilide **4** reacts with 3-hydroxybenzaldehyde in a one-pot Knoevenagel/epoxidation. Mild conditions using piperidine as catalyst were enough to promote the Knoevenagel condensation, and treatment with ^t^BuOOK and KF allowed the epoxidation in the same pot. Intermediate epoxide **5** undergoes an epoxide-arene cyclization by treatment with sulfuric acid resulting in 3-hydroxy-4-arylquinolin-2(1H)-one **6**. N-PMB derivative **6** is then transformed to the desired N-H deprotected quinolinone **1** when treated with trifluoroacetic acid (Scheme 2).

**Scheme 3:**
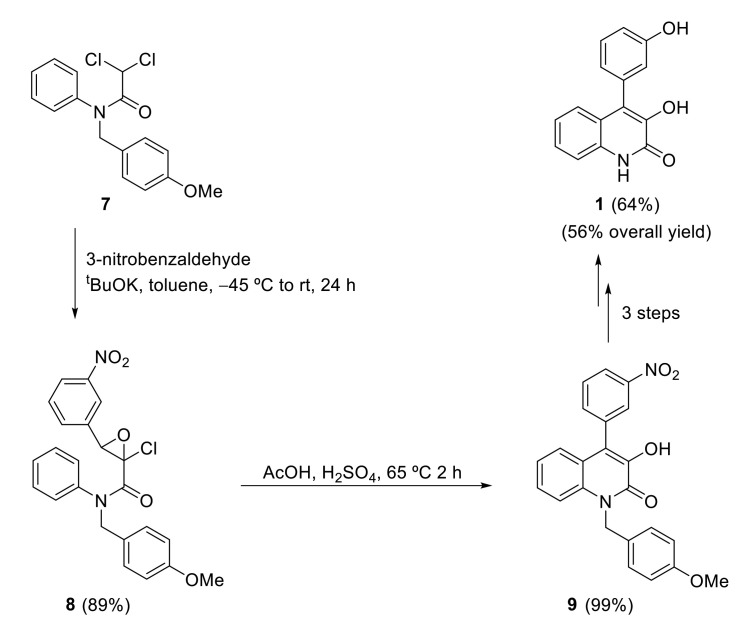
Synthesis of viridicatol by Mamedov’s group.

The synthesis of viridicatol also has recently been reported by other groups. As an example, Mamedov and coworkers developed a two-step process to 3-hydroxy-4-arylquinolin-2(1H)-ones starting with a Darzens condensation followed by an intramolecular Friedel–Crafts alkylation [18]. Dichloroacetanilide **7** reacts with 3-nitrobenzaldehyde in a Darzens condensation to produce epoxide **8**, which in an intramolecular Friedel–Crafts alkylation with acetic acid and sulfuric acid produces quinolinone **9**. Three further steps are required to obtain the natural product. Viridicatol **1** was synthesized in five steps from dichloroacetanilide **7** with 56% overall yield (Scheme 3).

### 2.2. Zooxanthellatoxin-A

Zooxanthellatoxin-A (ZT-A) is a novel polyhydroxypropylene isolated from a symbiotic marine alga *Simbiodinium* sp. Evidence indicates that this compound may work as a novel calcium agonist, inducing Ca^2+^-dependent aggregation in platelets and Ca^2+^-dependent muscle contraction in vascular smooth muscle cells. The final effect of calcium rinse induced by ZT-A seems mediated by activation of protein tyrosine kinase followed by phospholipase C-gamma 2 and protein kinase C, thus leading to MAP kinase activation and the ensuing release of arachidonic acid finally converted to TXA2 eliciting platelet aggregation in rabbits [19]. This compound also induced aorta contraction, and this effect was entirely dependent on the presence of extracellular Ca^2+,^ suggesting that ZT-A may promote Ca^2+^ entry into vascular smooth muscle cells, but the exact calcium entry channel activated by ZT-A remains unknown [20].

**Figure 3:**
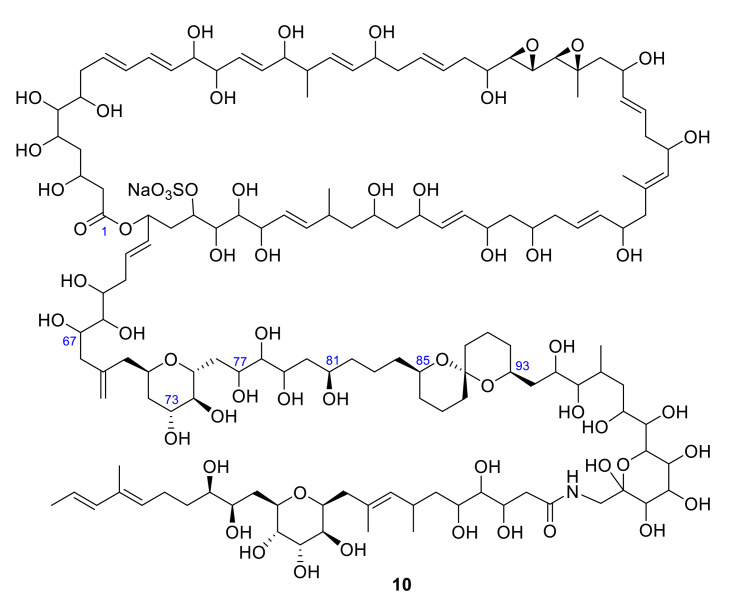
Structure of zooxanthellatoxin A **10** (ZT-A).

Its structure and absolute stereochemistry were determined by Nakamura and coworkers by synthesizing the fragments obtained during degradation experiments (Figure 3).

**Scheme 4:**
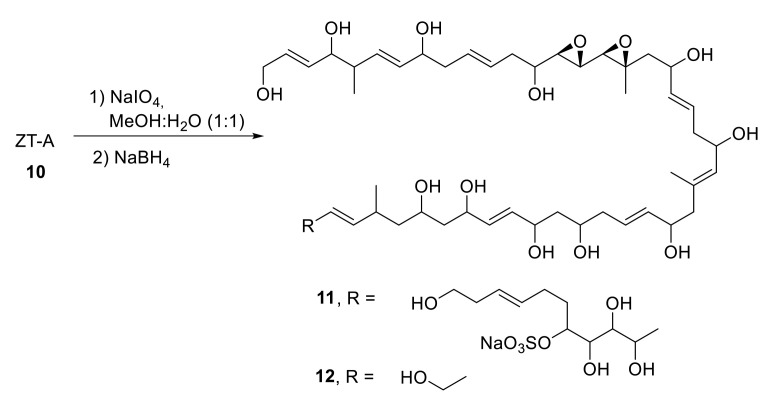
Degradation of ZT-A.

Treatment of ZT-A **10** with an excess amount of NaIO_4_, followed by reduction with NaBH_4,_ afforded fragments **11** and **12**, which possess a 1,3-diepoxide and a sulfate ester as characteristic functionalities (Scheme 4). DQF-COSY, 1D and 2D TOCSY (called HOHAHA at the time), HMBC, and NOE NMR spectra provided enough information to resolve the structure (carbon segments, olefinic proton signals and all connectivities) [21].

**Scheme 5:**
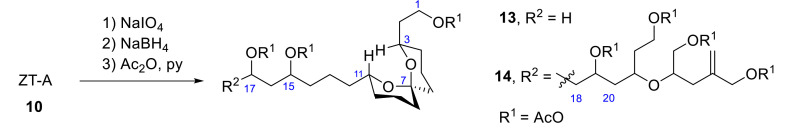
Obtention of spiroacetal alcohol peracetates **13** and **14** from ZT-A (**10**).

In 1996, spiroacetal alcohol peracetates **13** and **14** were obtained from ZT-A by oxidation with excess amounts of NaIO_4_, followed by reduction with NaBH_4_ and acetylation (Scheme 5) [22]. Their relative configuration was determined on the basis of NOE data and spin coupling constants.

**Scheme 6:**
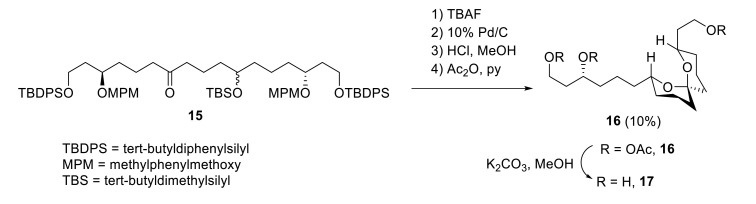
Synthesis of spiroacetal fragments **16** and **17** from intermediate **15**.

The synthesis of fragment **13** was achieved from advanced intermediate **15**. Two deprotection steps, spiroacetalization in the presence of HCl-MeOH and final acetylation, provided a stable spiroacetal **16** (R = AcO) in 10% yield (Scheme 6). The ^1^H NMR spectra of **16** and its triol (**17**) was similar to **13** and its triol, so it was assumed that the relative configuration of the spiroacetal unit was identical. Thus, the absolute stereochemistry of the degradation product **13** was determined to be 3*S*, 11*R* and 15*R*. These carbons correspond to C93, C85, and C81 of zooxanthellatoxin A.

**Figure 4:**
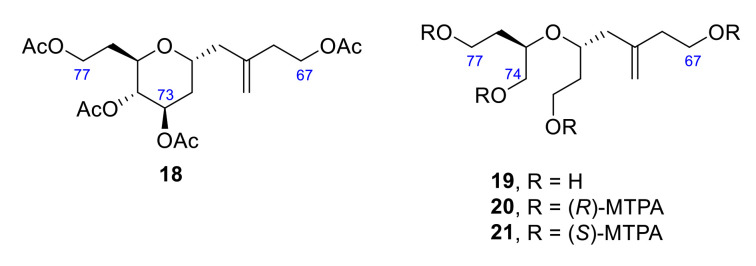
Structure of tetrahydropyran **18** and its acyclic derivatives **19**, **20** and **21**.

In 1998, oxidation of ZT-A with limited amounts of NaIO_4_, followed by reduction with NaBH_4_ and acetylation, provided the tetrahydropyran **18 [23]**, which was further deacetylated and subjected to NaIO_4_ oxidation to give **19** (Figure 4). Once acyclic tetraol **19** was synthesized, the stereochemistry of this synthetic product was established by transformation into its corresponding (*R*)- and (*S*)-α-methoxy-α-trifluoromethylphenylacetic (MTPA) esters (**20** and **21**). The spectra of the corresponding (*R*)-MTPA synthetic ester **19** and (*R*)-MTPA ester of natural degradation product were identical. Thus, the absolute configuration was confirmed to be 71*R*,73*R*,74*S*,75*R*.

**Scheme 7:**
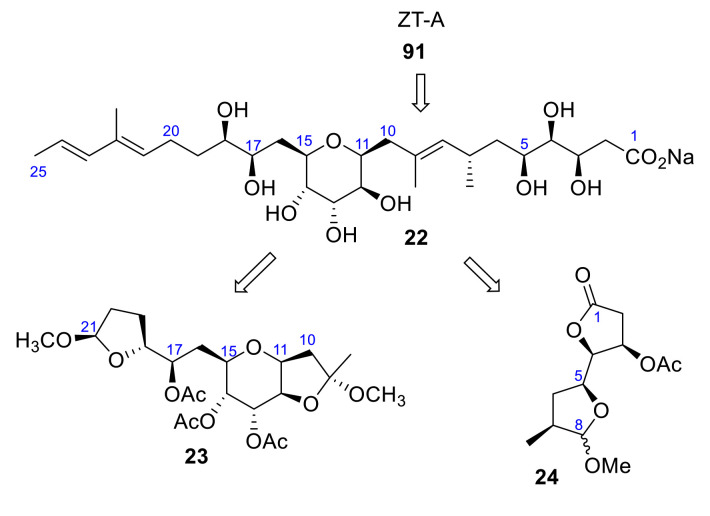
Structural fragments **23** and **24**.

To complete the structure assignment, just two fragments remained to be synthesized. The acid portion **22** is obtained by alkaline hydrolysis from the natural ZT-A. It was divided into two portions, **23** and **24,** that had to be synthesized in order to confirm the relative stereochemistry of this last part of the molecule (Scheme 7).

**Scheme 8:**
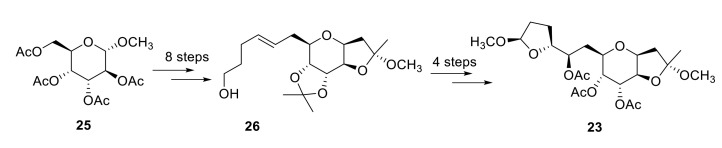
Synthesis of fragment **23**.

Fragment **23** was obtained from compound **25** in moderate yield after 12 steps (Scheme 8). By measuring the optical rotation and circular dichroism (CD) spectra, it was confirmed that the synthetic and the natural fragment were identical. Application of the Mosher method to dimethyl acetal derivative from **26** established the stereochemistry at C17. NOESY spectrum of **23** gave enough information to determine the configuration of C9 and C21 [24].

To confirm the stereochemistry of the rest of the carbons and the terminal carboxylic acid portion (C1–C8) of **22**, γ-lactones **24** were synthesized (Scheme 7). The similarity between the [α] _D_ values suggested that both fragments, natural and synthetic, had the same configuration, 3*R*,4*R*,5*S*,7*S*,11*S*,12*S*,13*R*,14*S*,15*R*,17*R*,18*R* [25].

More recently, a similar class of compounds, the zooxanthellamides (ZADs), were isolated. The zooxanthellamide Cs have improved biological properties with respect to zooxanthellatoxin A, showing a higher vasoconstrictive activity. Structure–activity relationship studies showed that the enormous macrolactone moiety is important for the observed properties. Nakamura’s efforts to the synthesis of zooxanthellatoxin A could be applied to the similar structures of this zooxanthellamides [26].

### 2.3. Massadine

Massadine is another marine drug reported to modulate Ca^2+^-signaling. This compound is a highly oxygenated alkaloid obtained from *Stylissa aff*, a marine sponge that has been described as a blocker of the enzyme geranylgeranyltransferase type I (GGTase I) with an IC50 in the low μM range [27]. Massadine decreases Ca^2+^ entry mediated by voltage-dependent Ca^2+^ channels in neuron-like PC12 cells loaded with the calcium probe fura2. This effect, in contrast to those of other compounds, was reversible [28]. Massadine holds two critical structures: a lipophilic, brominated side-chain involved likely in the good membrane solubility, and a hydrophilic, amino-imidazole substructure, possibly involved in the interaction with the channel itself. As intracellular Ca^2+^ overload is involved in neurotoxicity, the interaction with voltage-dependent Ca^2+^ channels may account for the neurotoxicity of massadine.

**Figure 5:**
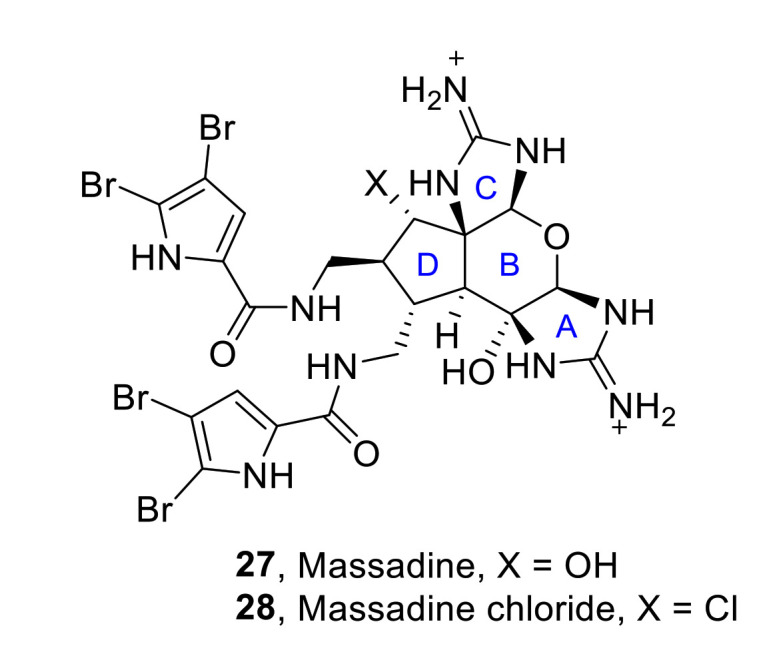
Structures of massadine **27** and massadine chloride **28**.

This hexacyclic marine natural product, belonging to the family of pyrrole-imidazole alkaloids, contains a highly congested and stereochemically rich tetracyclic core. Due to its complexity, in the last decades, various researchers have published numerous articles describing the partial or total synthesis of massadine **27** and massadine chloride **28** (Figure 5).

**Scheme 9:**
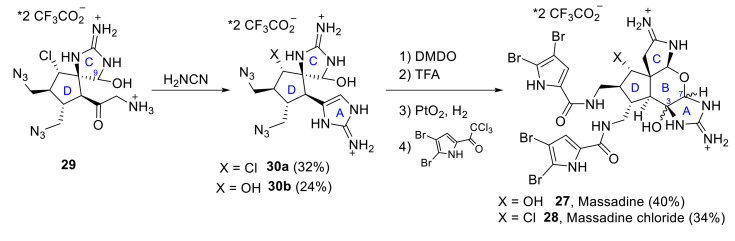
Baran’s total synthesis of massadine **27** and massadine chloride **28**.

The first total synthesis of massadine **27** and massadine chloride **28** was reported by P. S. Baran et al. in 2008 [29,30]. Despite the constitutionally isomeric nature between massadine chloride and axinellamines, a rearrangement was not observed under any of the conditions tested. Thus, it was necessary to propose a different synthetic approach, which key intermediate is the spirocycle **29**. Thus, starting from intermediate **29,** the construction of the different heterocyclic units of massadines was achieved in sequential steps. First of all, the formation of the desired 2-aminoimidazole **30a** (32%) (along with its hydroxy analog **30b** (24%)) was performed by reaction of **29** with cyanamide. The subsequent formation of the tetrahydropyranyl ring was achieved by oxidation with dimethyldioxirane (DMDO) followed by acid-mediated closure with neat trifluoroacetic acid (TFA). Finally, reduction of the azide moieties, followed by reaction with bromopyrrole, afforded the final desired natural products, massadine **27** (H, OH = α) and massadine chloride **28** (H, OH = α), in 40% and 34% yield, respectively and their epimers (H, OH = β), although these epi-series have not been found in nature (Scheme 9).

**Scheme 10:**
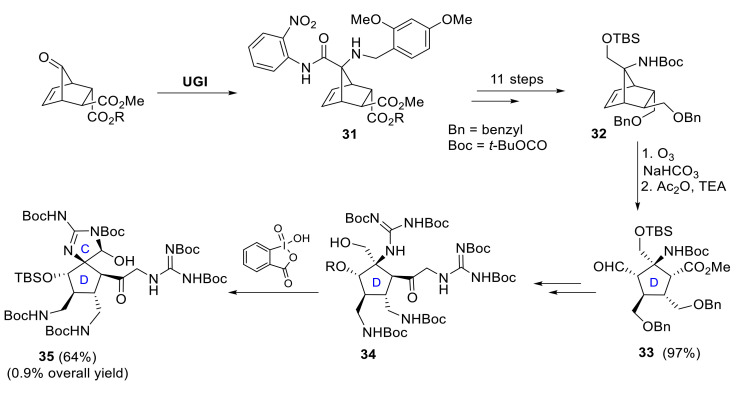
Synthesis of norbornene derivative **31** by Ugi-4-component reaction and further elaboration to CD-ring subunit **35**.

A different approach to synthesize the functionalized core structure **35** of massadine in an enantioselective manner has been developed by E. Carreira et al. Two key steps are involved in the successful construction of the D-ring subunit: an Ugi-4-component reaction affords intermediate **31**, followed by 11 steps to reach the appropriately functionalized norbornene derivative **32 [31,32]**, and end-group-differentiating ozonolysis that yields compound **33**. The corresponding synthesis of the spirocyclic CD-ring subunit **35** of massadine was then achieved by reaction of intermediate **34** with 2-iodoxybenzoic acid. Carreira and coworkers carried out the synthesis of this advanced intermediate in a sequence of a total of 37 linear steps and with an overall yield of about 0.9% (Scheme 10) [33].

**Scheme 11:**
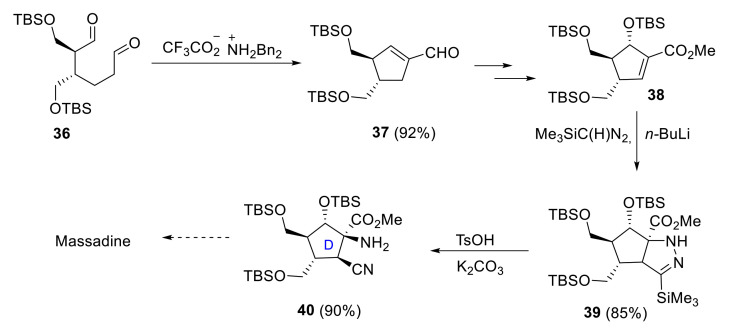
Synthesis of the D-ring subunit **36** of massadine proposed by Lee.

Lee and coworkers proposed a different strategy for synthesizing the carbocyclic core **40** of massadine [34]. Key steps involved a stereoselective (3 + 2) cycloaddition and a protonolytic N-N bond cleavage in obtaining the tertiary α-amino group and the β-cyano group in a *cis* arrangement. The preparation of functionalized cyclopentane **40** started with the formation of the cyclopentene carboxaldehyde **37** by the reaction of hexanodial **36** with a catalytic amount of dibenzylammonium trifluoroacetate. The key α-amino-β-cyanation protocol was achieved by the reaction of advanced intermediate **38** with lithiumtrimethylsilyldiazomethane and subsequent acid-mediated N-N bond cleavage (Scheme 11).

**Scheme 12:**
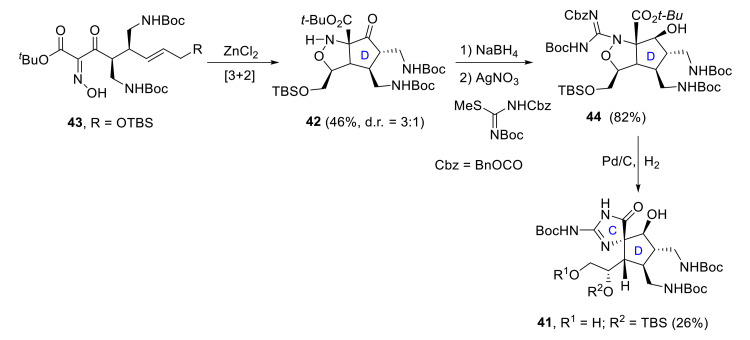
Cannon’s synthesis of the CD-ring subunit **41** of massadine.

In 2018, Cannon reported an enantioselective route to the CD-bicycle of massadine **41 [35]**. His strategy consisted of the formation of the cyclopentanone moiety **42** through a thermal nitrone dipolar cycloaddition of intermediate **43**. Differentially substituted guanidine **44** was prepared by reduction of the ketone **42** followed by silver-mediated guanylation of the isoxazolidine. Final formation in good yields of CD-bicycles **41** involved a double hydrogenolysis process with different effects: the hydrogenolysis of the N-O bond opened the isoxazolidine ring, while the hydrogenolysis of the CBz protecting group induced the spirocyclic ring closure to form C ring (Scheme 12).

### 2.4. Crambescidin 816

Crambescidin 816 (Cramb816) is a member of a family of guanidine alkaloids obtained from *Crambe crambe*, a Mediterranean marine sponge. This compound with antitumoral activity has several biological effects related to the modulation of Ca^2+^ signaling. In the first place, electrophysiological evidence obtained in neuroblastoma cells and neurons indicates that Cramb816 inhibits Cav1 or L-type, voltage-dependent Ca^2+^ channels with higher potency than dihydropyridines such as nifedipine. Cramb816 inhibited partially also voltage-dependent Na^+^ channels. These effects may account for the inhibition of synaptic transmission in the central nervous system [36]. Consistently with these effects, Cramb816 also prevented the Ca^2+^-dependent ileum contraction induced by acetylcholine at very low concentrations [37]. Paradoxically, Cramb816 induces a dose-dependent increase in intracellular Ca^2+^ and reduces viability in neurons. This effect is prevented by glutamate receptor antagonists suggesting that Cramb816 activates glutamate receptors, thus promoting Ca^2+^ overload and cell death in cortical neurons [38].

**Figure 6:**
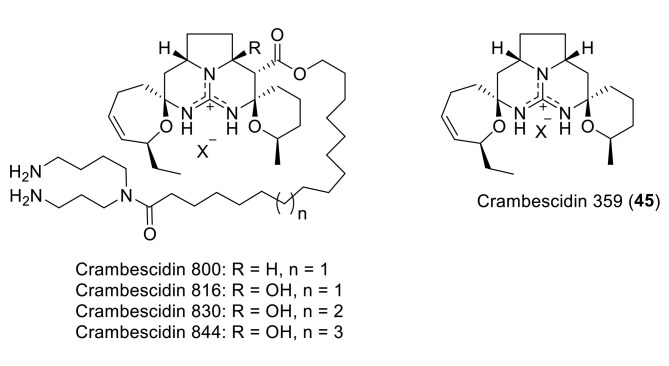
The crambescidin family.

The crambescidin family contains a unique pentacyclic guanidine unit and a spermidine unit linked by a linear long-chain fatty acid (Figure 6). In 2000, the first derivative compound lacking the spermidine unit was isolated from the marine sponge of the genus *Monanchora* by Breakman et al. under the name of crambescidin 359 (**45**) [39].

**Scheme 13:**
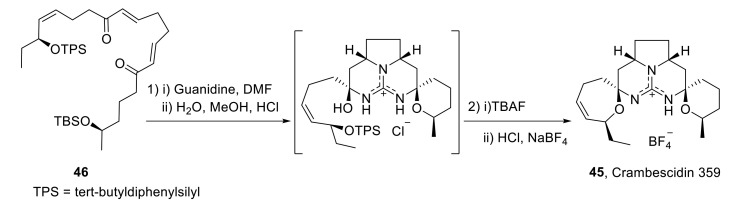
Moore’s synthesis of crambrescidin 359 (**45**).

No total synthesis of crambescidin 816 has been reported so far, although various approaches to the crambescidin core present in crambescidin 359 have been described. Thus, in 1999, shortly before the isolation of crambescidin 359, C.G. Moore achieved its total synthesis during his PhD, although the work was published in 2007 [40]. The key step for the formation of the core tetracycle implies the reaction of bis-enone **46** with guanidine. Subsequent treatment with TBAF and acid-mediated cyclization gave the desired pentacycle **45** in 18% overall yield (Scheme 13).

**Scheme 14:**
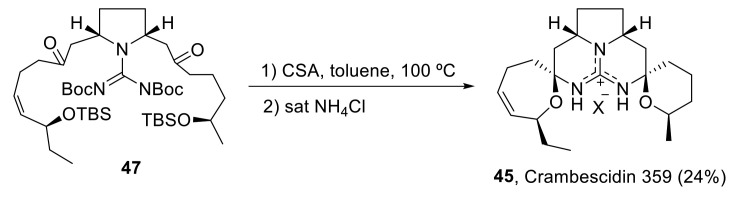
Total synthesis of crambescidin 359 (**45**).

In 2003, the first total synthesis of crambescidin 359 (**45**) was published [41], in which the key step consists of the double *N,O*-acetalization of the guanylated diketone **47** (Scheme 14).

**Scheme 15:**
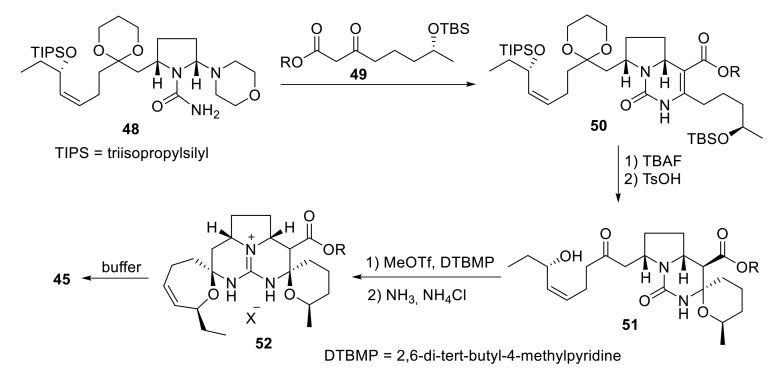
Overman’s synthesis of crambescidin 359 (**45**).

Two years later, Overman et al. reported the total enantioselective synthesis of crambescidin 359 (**45**) in 29 total steps and 2.8% overall yield. The construction of the pentacyclic core was accomplished in sequential steps such as Biginelli condensation of aminal **48** with β-ketoester **49**; acid-mediated spirocyclization of the deprotected pyrrolopyrimidine **50** and transformation of tricyclic urea **51** into the methyl pseudourea derivative, followed by reaction with ammonia, gave crambescidin analogs of type **52**, which afford crambescidin 359 (**45**) after decarboxylation (Scheme 15) [42].

### 2.5. Maitotoxin

Maitotoxin (MTX) is an extremely potent toxin that is produced by *Gambierdiscus toxicus*, a dinoflagellate species. This toxin induces *ciguatera fish poisoning*, a human intoxication syndrome. MTX promotes Ca^2+^ influx at extremely low concentrations in the pM range. This effect seems mediated by the activation of Ca^2+^-permeable, non-selective channels belonging to the large superfamily of transient receptor potential channels (TRP channels). Ca^2+^ and Na^+^ influx induced by activation of these channels may lead to plasma membrane depolarization, which, in excitable cells, may, in turn, recruit voltage-gated Ca^2+^ channels leading to massive Ca^2+^ influx in neurons, muscle or endocrine cells. Recent evidence shows that siRNA-mediated silencing of the TRPC1 channel, the first member of the canonical family of channels, abolishes Ca^2+^ influx induced by MTX, thus suggesting that MTX targets and activates TRPC1 channels [43].

**Figure 7:**
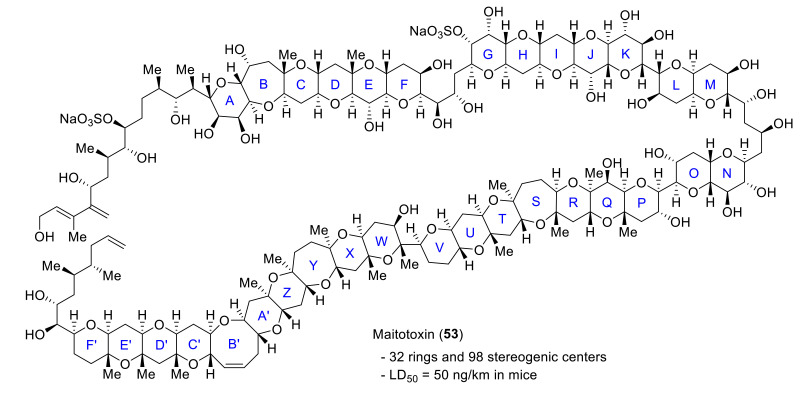
Structure of maitotoxin (**53**).

Maitotoxin is the largest among the secondary metabolites isolated and characterized up to date. This fact makes it an inspiration to many synthetic chemists. On the other hand, small fragments have been synthesized in order to support the absolute stereochemistry assigned in its characterization and as an approach to its total synthesis. In addition, the efforts towards the synthesis of this huge molecule have pushed the limits of organic synthesis, and many new synthetic methods have been developed along the way (Figure 7).

If there is one group that stands out for their efforts towards the synthesis of this challenging molecule is the Nicolaou group. Relying on processes like the Noyori reduction/Achmatowicz rearrangement, hydroxy dithioketal cyclization or SmI_2_-mediated ring closure, they have synthesized the ABCDEFG, QRSTU [44], WXYZA’ [45] and C’D’E’F’ [46] ring systems. These advances in the synthesis of large maitotoxin domains were reviewed up to 2011 [47]. More recently, in 2014, Nicolaou reported the synthesis of the QRSTUVWXYZA′ system of maitotoxin [48]. Comparison of ^13^C NMR data supported the original structural assignment in all cases.

**Scheme 16:**
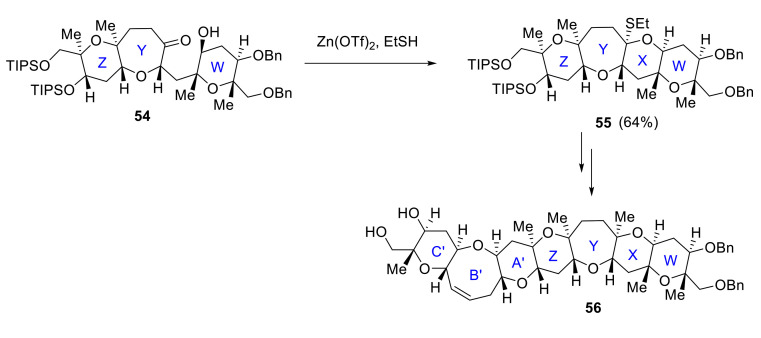
Formation of the WXYZA’B’C’ ring by Oishi group.

Another group that has worked towards the synthesis of maitotoxin is the Oishi group. In 2008, Oishi reported a convergent synthesis of the C’B’A’ZYXW ring **56**, relying on acetal or thioacetal formation as the key cyclization step [49]. For instance, the preparation of the ZYXW tetracyclic intermediate **55** relies on the formation of a mixed thioacetal ring by the reaction of hydroxyketone **54** with ethanethiol in the presence of Zn(OTf)_2_ (Scheme 16).

**Scheme 17:**
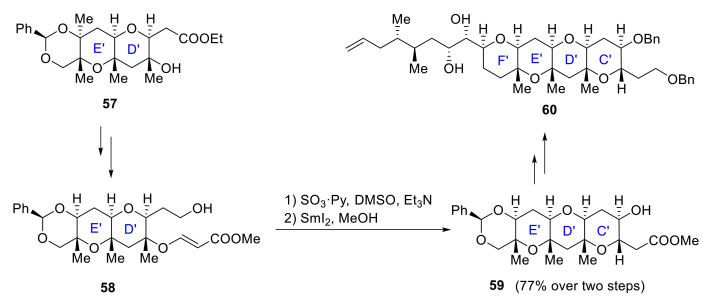
Stereoselective formation of the C’D’E’F’ ring by Oishi group.

In 2014, Oishi reported the stereoselective synthesis of the F’E’D’C’ ring system of maitotoxin starting from E’ ring. E’D’ ring system **57** is formed by SmI_2_-induced cyclization of E’ ring, and E’D’C’ ring **59** formation follows the same procedure, starting from **58** (Scheme 17). The side-chain fragment was installed via Suzuki−Miyaura cross-coupling, and F’ ring formation was achieved by a Pd(II)-catalyzed cyclization yielding the desired compound **60** [50].

**Scheme 18:**
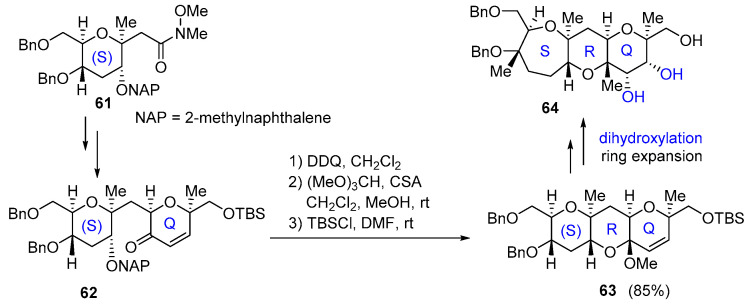
Stereoselective formation of the QRS ring by Oishi group.

Finally, in 2014 Oishi also proposed the stereoselective synthesis of the SQR ring of maitotoxin based on the formation of five contiguous stereogenic centers on the Q ring. The synthesis starts from (S) ring **61**, which is transformed in several steps to intermediate **62**. Removal of NAP group with DDQ, followed by the conversion of the hydroxy ketone to methyl acetal with (MeO)_3_CH, produces (S)RQ ring **63**. After highly diastereoselective dihydroxylation and ring expansion of a six-membered to a seven-membered ring ketone, final SRQ ring **64** is obtained (Scheme 18) [51]. Similar strategies have also been applied to the synthesis of the LMNO ring system [52].

Other groups have also made great efforts in the synthesis of different parts of this amazing molecule. In 2008, Nakata published a series of papers describing the synthesis of C’D’E’F’ [53], WXYZA’ [54] and BCDE [55] ring systems relying on SmI_2_ reductive cyclization. More recently, in 2017, Saito and Nakata synthesized the enantiomer of the ZA′B′C′D′-ring system of maitotoxin through a convergent strategy [56].

### 2.6. Yessotoxin

**Figure 8:**
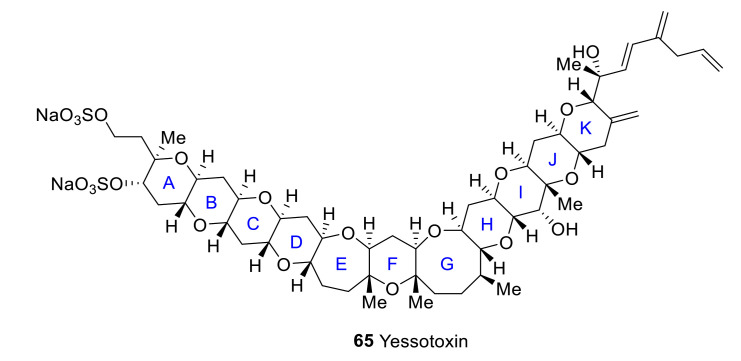
Structure of yessotoxin.

Yessotoxin (YTX) **65** is a polyether toxin related to ciguatoxins and produced by different dinoflagellates, particularly *Lingulodinium polyedrum* and *Gonyaulax spinifera* (Figure 8). This toxin has been reported to induce also Ca^2+^ influx in different cell types, including lymphocytes, and to promote neurotoxicity in cultured cerebellar neurons [57]. Ca^2+^ entry induced by yessotoxin is prevented by Nifedipine and SKF96365, a well-known blocker of L-type, voltage-gated Ca^2+,^ and other types of channels. Yessotoxin may modulate L-type Ca^2+^ channels and also other types of Ca^2+^ channels [58]. Yessotoxin also induced Ca^2+^ influx and apoptosis in human hepatocellular carcinoma (Bel7402) cells, and this effect was blocked by external Ca^2+^ chelation and nifedipine, thus suggesting that YTX-induced apoptosis is mediated by activating a Ca^2+^ entry pathway and Ca^2+^ overload [59].

**Scheme 19:**
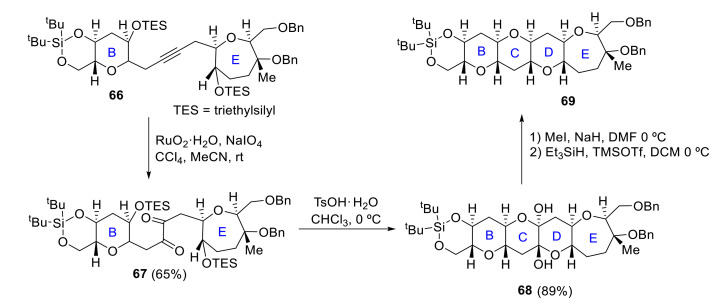
Mori’s synthesis of BCDE ring.

In 2002, Mori reported a convergent synthesis of the BCDE ring [60]. B and E rings are coupled with an alkyne moiety via alkylation with acetylene. 1,2-diketone **67**, obtained by ruthenium oxidation of intermediate alkyne **66**, is treated with TsOH to produce tetracyclic dihemiketal **68**. *O*-methylation of the two hemiketals hydroxyl groups **68** and reductive etherification results in the formation of BCDE ring system **69** of yessotoxin (Scheme 19). Later, similar methodologies were applied to synthesize the bigger CDEFGHIJ fragment [61].

**Scheme 20:**
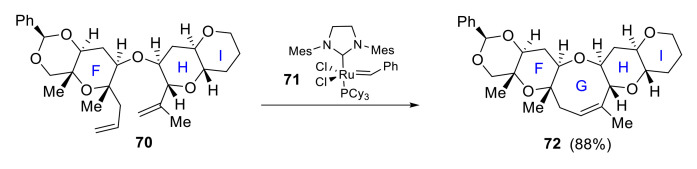
Kadota’s synthesis of FGHI ring.

Kadota reported in 2006 a convergent synthesis of FGHI ring system **72** in which the key step in the construction of ring G by ring-closing metathesis of diene **70** with second-generation Grubbs catalyst **71** (Scheme 20) [62].

**Scheme 21:**
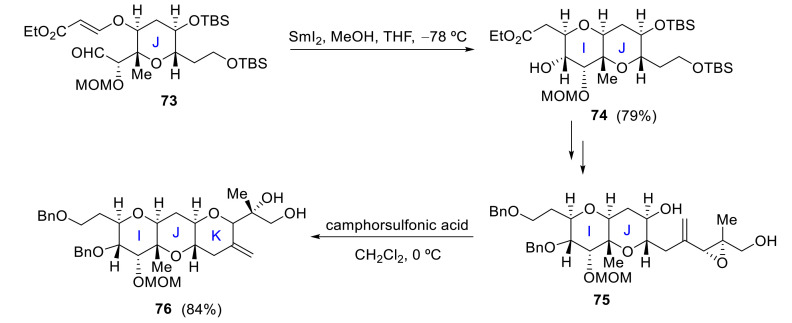
Kadota’s approach to the IJK ring system of yessotoxin.

In parallel, Kadota also reported a stereocontrolled synthesis of IJK ring **76**, where SmI_2_- and acid-induced cyclization is the key ring-closure process. Thus, SmI_2-_mediated cyclization of **73** produces IJ ring **74** while the K ring is later constructed via acid-catalyzed cyclization of epoxy alcohol **75** (Scheme 21) [63].

**Scheme 22:**
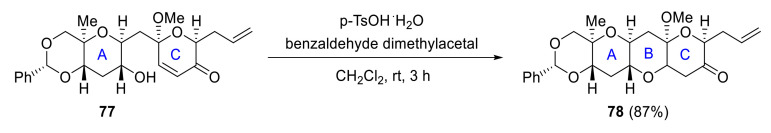
Oishi’s synthesis of the ABC ring.

**Scheme 23:**
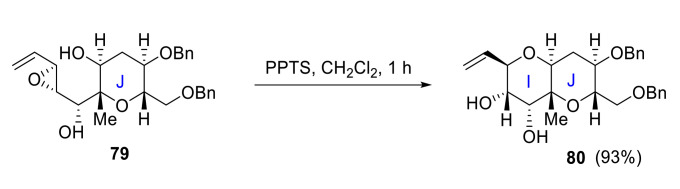
Oishi’s synthesis of IJ ring.

In 2006, Oishi reported the synthesis of ABC and IJ rings. Cyclization of hydroxyenone **77** in the presence of pTsOH produces ABC ring system **78** (Scheme 22), while IJ ring fragment **79** is synthesized starting from the J ring. Acid-catalyzed 6-*endo* cyclization of the vinyl epoxy alcohol **79** results in IJ ring **80** in 93% yield (Scheme 23) [64].

**Scheme 24:**
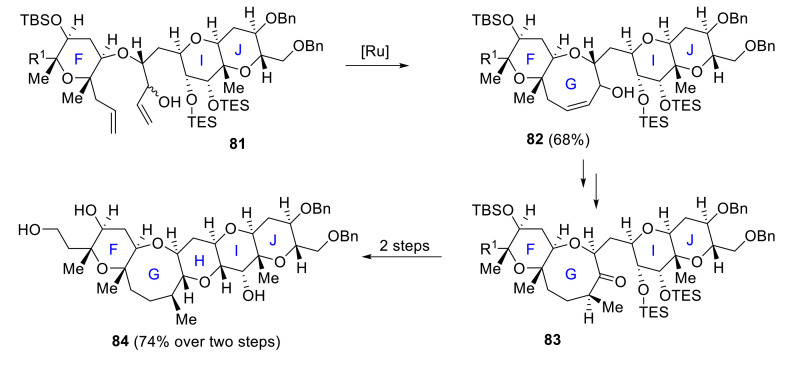
Oishi’s synthesis of the FGHIJ ring.

In 2005, Oishi reported a route to the FGHIJ fragment **84** [65], and in 2008 he finally achieved a highly convergent synthesis of the A–J ring system [66]. The main features for the construction of the pentacyclic F–J rings are a ring-closing metathesis process (**81** to **82**) and the formation of a cyclic acetal from a hydroxy ketone (**83** to **84**) [67] (Scheme 24).

**Scheme 25:**
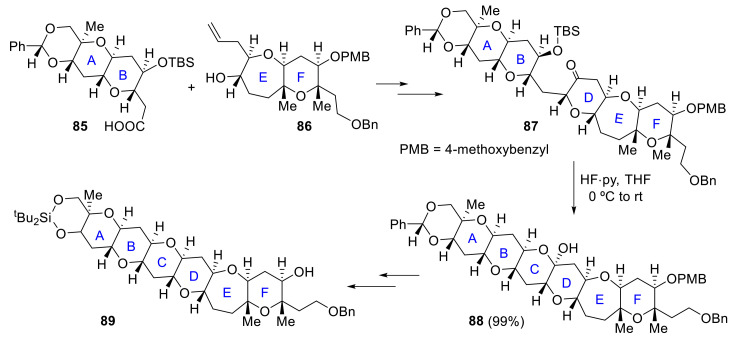
Zhang and Rainier’s approach to the ABCDEF ring system of yessotoxin.

**Scheme 26:**
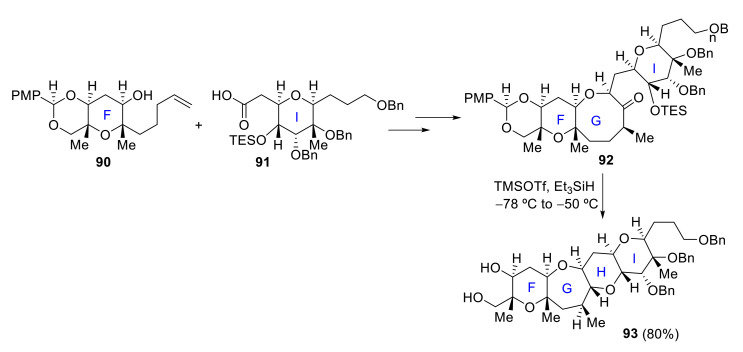
Zhang and Rainier’s approach to the FGHI ring system of yessotoxin.

In 2016, Zhang and Rainier proposed a synthesis for ABCDEF and FGHI rings. Fragments AB **85** and EF **86** were easily obtained from known precursors previously synthesized for the total synthesis of brevenal [68]. Ring C is formed by removal of the TBS group of intermediate **87** and intramolecular acetalization, affording hemiketal **88**. Final ABCDEF ring **89** was produced from **88** in a few more steps (Scheme 25). Regarding the FGHI fragment, F ring **90** and I ring **91** are coupled by Yamaguchi esterification. After some steps, including olefinic-ester cyclization to obtain the G ring, the final intermediate **92** is obtained. The last H ring is then crafted via reductive cyclization of this TES-protected hydroxy ketone **92** with TMSOTf/Et_3_SiH, affording the desired FGHI ring system **93** (Scheme 26) [69].

Some groups have also developed different methodologies to synthesize small fragments of this marine drug relying on transition metal catalysis. Regarding palladium catalysis, it can be applied to the construction of the AB ring system as demonstrated by Hirai [70] and Jamison [71] or to the KJ ring system as shown by Yokoyama [72]. Other metals like ruthenium have shown their applicability in this synthesis, like in Trost’s approach to the BCD ring system by a ruthenium-catalyzed cycloisomerization reaction [73].

### 2.7. Palytoxin

Palytoxin (PTX) is a highly thermostable, polyhydroxylated and partially unsaturated compound with as much as eight double bonds within a long carbon chain. It is produced by *Palythoa* corals and *Ostreopsis* dinoflagellates, as well as by bacteria living in these organisms. It can also be isolated from other living organisms close to palytoxin producing organisms like sponges, mussels, starfish and cnidaria. PTX is among the most potent toxins found in nature. PTX produces severe vasoconstriction, disrupts cardiac function, and it is highly lethal to mice. PTX strongly inhibits the Na^+^/K^+^ATPase pump leading to a loss of ion gradients and plasma membrane depolarization that triggers activation of voltage-dependent ion channels, including voltage-dependent Ca^2+^ channels, reverse operation of the Na^+^/Ca^2+^ exchanger and induces Ca^2+^ overload. Most of these effects are prevented by ouabain [74]. As a consequence, PTX induces the release of neurotransmitters from motor nerve terminals [75].

PTX also elicits the release of glutamate from cerebellar granule cells leading to their depletion as well as prostaglandins from rat aorta with intact endothelium [76]. Finally, PTX is also an atypical tumor promoter on mouse skin initiated by irritants, probably because of its proinflammatory action [77].

**Figure 9:**
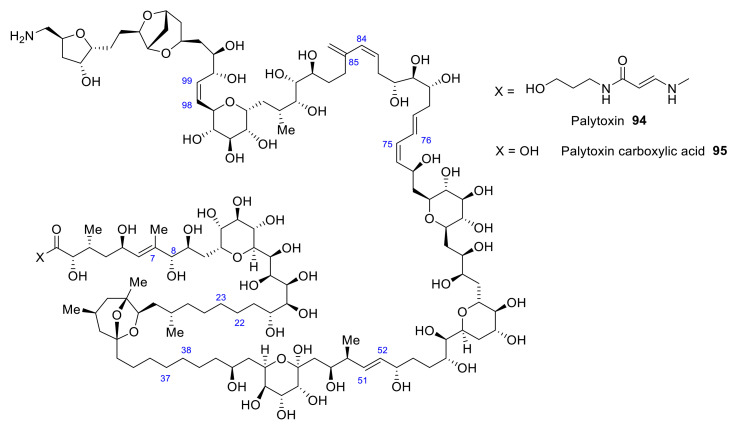
Structure of palytoxin and palytoxin carboxylic acid.

Initial studies of palytoxin focused on determining its stereochemistry, relying on the synthesis of some of its degradation products [78]. Thus, the structure of palytoxin **94** was elucidated, as depicted in Figure 9. Due to its size and structural and stereochemical complexity, palytoxin is one of the most challenging compounds in the field of total synthesis.

**Scheme 27:**
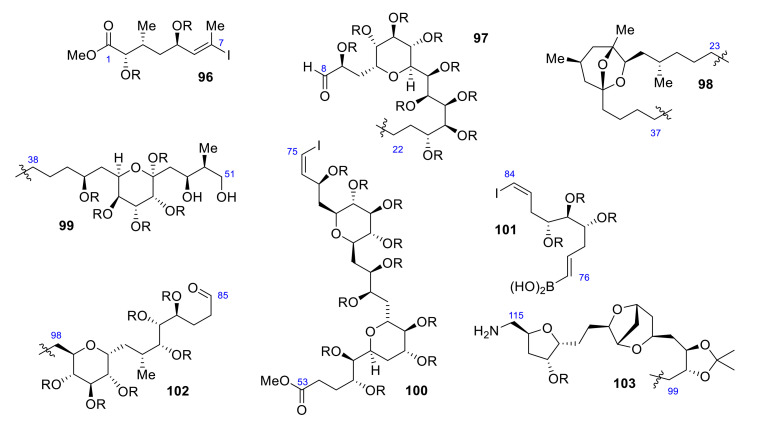
Retrosynthesis of palytoxin carboxylic acid.

Seven years after its isolation, Kishi reported its first total synthesis [79]. He focused on obtaining eight different segments (Scheme 27) whose coupling produced palytoxin carboxylic acid **95**. Palytoxin carboxylic acid retrosynthesis results in a division in eight fragments: C1-C7 (**96**), C8-C22 (**97**), C23-C37 (**98**), C38-C51 (**99**), C52-C75 (**100**), C76-C84 (**101**), C85-C98 (**102**), C99-C115 (**103**).

Before proceeding to the coupling of the fragments, they needed to be synthesized. Each segment requires a different coupling, being some of them relatively straightforward, while others are more challenging.

Coupling between C22-C23 and C37-C38 goes via Wittig reaction followed by hydrogenation, so a C8-C51 chain is synthesized (**104**).

**Scheme 28:**
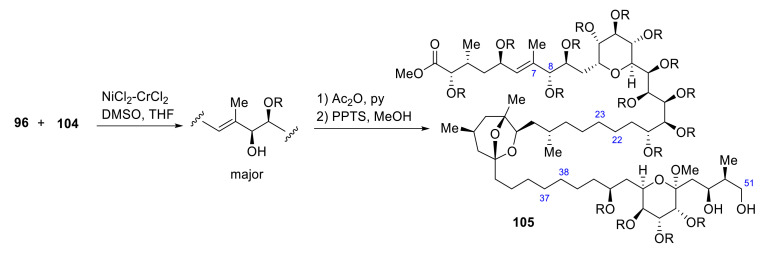
Synthesis of fragment **105** of palytoxin carboxylic acid.

A Ni(II)/Cr(II)-catalyzed coupling reaction was then used to bond **96** with **104**. After acetylation and hydrolysis of the acetonide, **105**, left half of **95**, is obtained (Scheme 28).

**Scheme 29:**
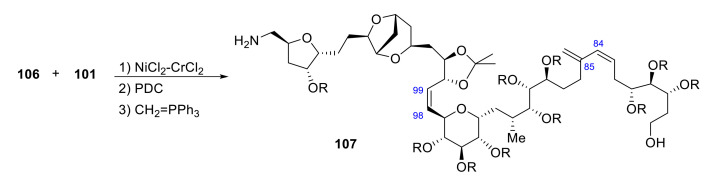
Synthesis of fragment **107** of palytoxin carboxylic acid.

Segments **102** and **103** are coupled by a Wittig reaction producing the C98-C99 olefinic bound. **106** reaction with **101**, relying on Ni(II)/Cr(II) catalysis, followed by PDC oxidation and Wittig olefination, produces C84-C85 olefinic bond (Scheme 29).

**Scheme 30:**
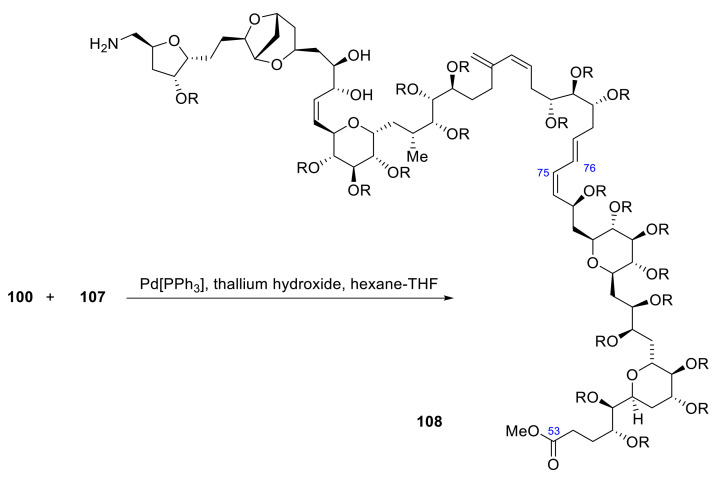
Synthesis of fragment **108** of palytoxin carboxylic acid.

**107** reacts with **100** producing C75-C76 coupling by a Suzuki reaction (Scheme 30).

**Scheme 31:**
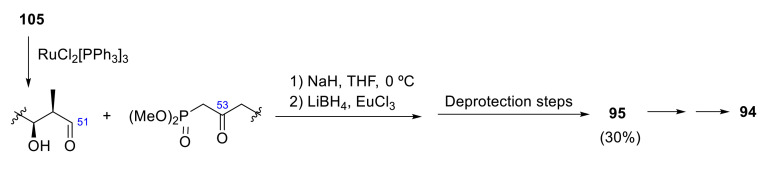
Synthesis of palytoxin.

Once the initial eight fragments have been coupled to form **105** and **108**, the C51-C52 olefinic bond produces the coupling of both fragments to form **95**. **105** is oxidized with RuCl_2_[PPh_3_] _3_ to form an aldehyde, which is coupled with the anion generated from the ketophosphonate derived from methyl ester **108** to form an α,β-unsaturated ketone in 80–90% yield. After, C53 ketone is reduced to an alcohol by lithium borohydride in the presence of a lanthanide salt (EuCl_3_). As numerous protection groups have been used, a deprotection step is required to obtain the final product. All deprotection steps produce a 30% overall yield of the carboxylic acid **95** (Scheme 31). Finally, a functional group transformation of the carboxylic acid of palytoxin carboxylic acid **95** to the required N-acyl vinylogous urea (via acid-catalyzed lactonization, followed by addition with the corresponding amine bearing a phenylselenide group and final oxidative elimination of the selenide) provides palytoxin **94** [80].

After this first total synthesis, some other approaches have been developed, being the main objective of most of them the quantitative stereospecific synthesis of a fragment. For instance, Still’s approach pursues the synthesis of C30-C43 fragments by a stereospecific macrocyclically controlled remote asymmetric induction process [81]. On the other hand, Nelson’s objective was to synthesize the C58-C71 segment of palytoxin [82]. Since this fragment is close to having a C2-symmetry, being the features that break symmetry the absence of a hydroxyl group in C59, and different (2,6) relative configurations of the two tetrahydropyran rings, this method proposed a two-directional synthesis for a C2-symmetrical fragment, which will be later modified in order to obtain functionalized tetrahydropyrans in a stereoselective manner.

## 3. Heterocyclic Marine Drugs with a Direct Effect over Cytoskeleton

### Latrunculin A

Latrunculin A is a member of the family of natural products and toxins termed latrunculins. At low μM concentrations, it binds actin monomers preventing their polymerization, thus resulting in the disruption of actin filaments of the cytoskeleton [83]. This effect may also result in the modulation of Ca^2+^ channels, specifically intracellular Ca^2+^ channels, as the inositol trisphosphate (IP_3_) receptor, a highly relevant, ligand-gated Ca^2+^ channel located in the membrane of the ER and responsible for agonist-induced Ca^2+^ release from intracellular stores. Accordingly, latruculin A may induce Ca^2+^ release from the ER, then the depletion of intracellular Ca^2+^ stores may activate store-operated Ca^2+^ channels and secondary Ca^2+^-dependent Na^+^ entry, which may promote membrane depolarization. These effects are similar to those induced by cytochalasin D, a well-known actin disruptor. Latruculin A-elicited depolarization consisted of a positive shift in Vm, which reached the threshold of activation of voltage-dependent Ca^2+^ channels, thus triggering an action potential. In Ca^2+^-free medium latruculin A lacked the ability to induce action potentials, while it was abolished upon removal of external Na^+^. Moreover, membrane depolarization was prevented by pre-injection of BAPTA and heparin, but not ryanodine [84]. Actin polymerization is involved in many different cell functions that are disrupted by latrunculin A. For instance, actin polymerization is required for clathrin-coated vesicle maturation of lamprey synapses. It is also very important for myogenic constriction in cerebral arteries [85]. Another example is egg fertilization that requires Ca^2+^ waves that depend on an intact cytoskeleton [86].

**Figure 10:**
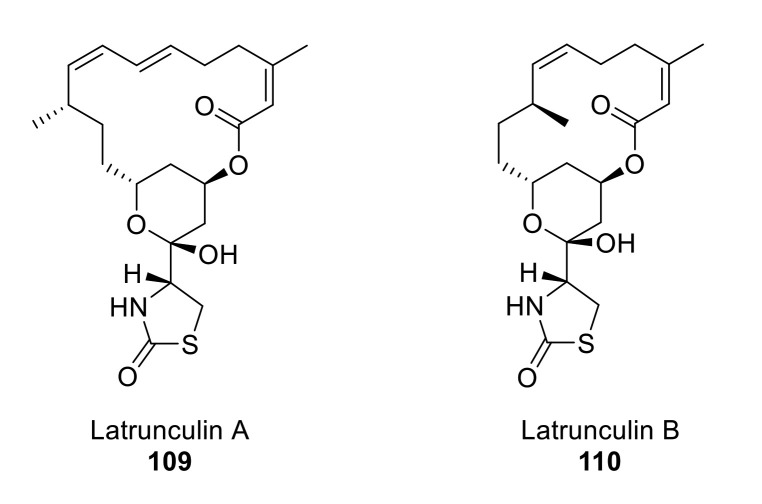
Structures of latrunculin A and B.

Latrunculins A and B (Figure 10) were first isolated and characterized by Kashman from the Red Sea sponge *Latrunculia magnifica* (Keller) [87].

**Scheme 32:**
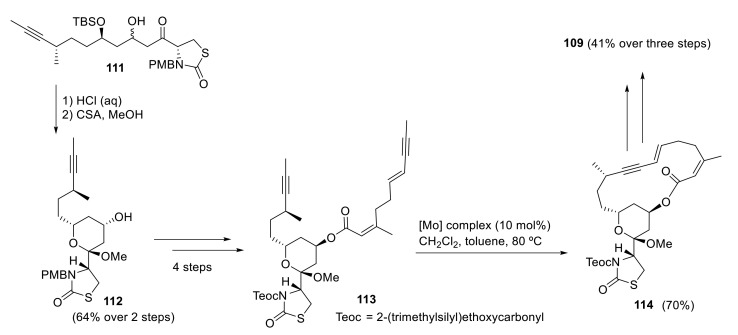
Fürstner’s synthesis of latrunculin A.

Fürstner and coworkers designed a versatile synthetic route to different members of the latrunculin family through ring-closing enyne-yne metathesis [88,89,90]. Treatment of intermediate **111** with aqueous HCl deprotected the TBS group and also affected the cyclization into hemiacetal, which was methylated with catalytic CSA and methanol to give **112**. Ring-closing alkyne metathesis (RCAM) of intermediate **113** (obtained from **112** in four steps) produces the highly strained 16-membered cycle **114** in an enyne-yne metathesis catalyzed by a molybdenum complex. Compound **114**, the smallest ring size that has been synthesized by an enyne-yne metathesis, forms **109** in three further steps in 41% overall yield [90] (Scheme 32). The total synthesis of latrunculin A was achieved in 15 linear steps over 1.7% overall yield.

As latrunculin A differs from other members of its family in the presence of a conjugated diene, White’s convergent synthetic approach proposes the formation of an acyclic *E,Z*-diene formed by Wittig reaction of an *E*-ylide with an aldehyde [91].

**Scheme 33:**
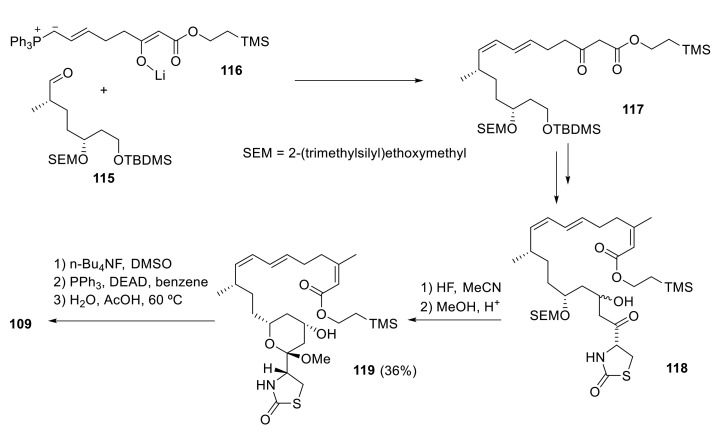
White’s synthesis of latrunculin A.

Thus, the reaction of aldehyde **115** with *E*-ylide **116** gives *E,Z*-diene **117**, which is further elaborated to produce intermediate aldol **118** in five steps. The final steps to **109** from **118** are quite straightforward. Treatment with HF and methanol allows selective deprotection of SEM group and subsequent cyclization, thus closing the desired tetrahydropyran ring through the formation of a methyl ketal. Finally, **119** is transformed to **109** in three additional steps (Scheme 33).

**Scheme 34:**
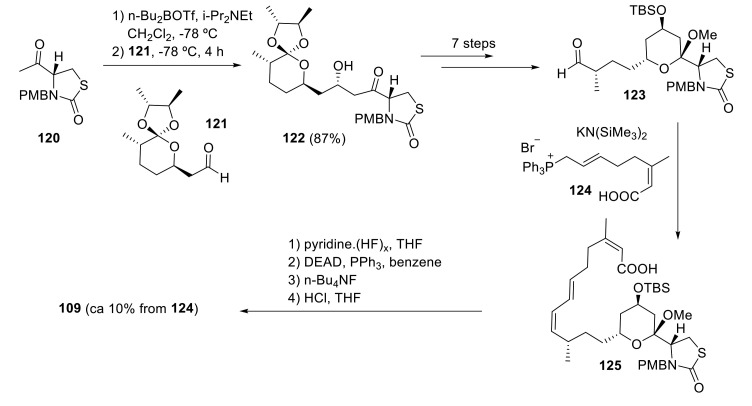
Smith highly convergent synthesis of latrunculin A.

Smith and coworkers designed a short and highly convergent total synthesis of latrunculin A (**109**), where the longest linear sequence is 17 steps [92]. Treatment of **120** with n-Bu_2_BOTf and i-Pr_2_NEt followed by reaction with aldehyde **121** produces **122** (major isomer), which is then transformed to **123** in 22% yield over seven steps. Wittig coupling of aldehyde **123** with ylide **124** furnishes a *cis-trans* diene **125** that in four steps produces **109** (Scheme 34).

Building block **123**, as well as an intermediate for the synthesis of latrunculin A **109**, is useful for the synthesis of other latrunculin family members [93], as they share a common structure.

**Scheme 35:**
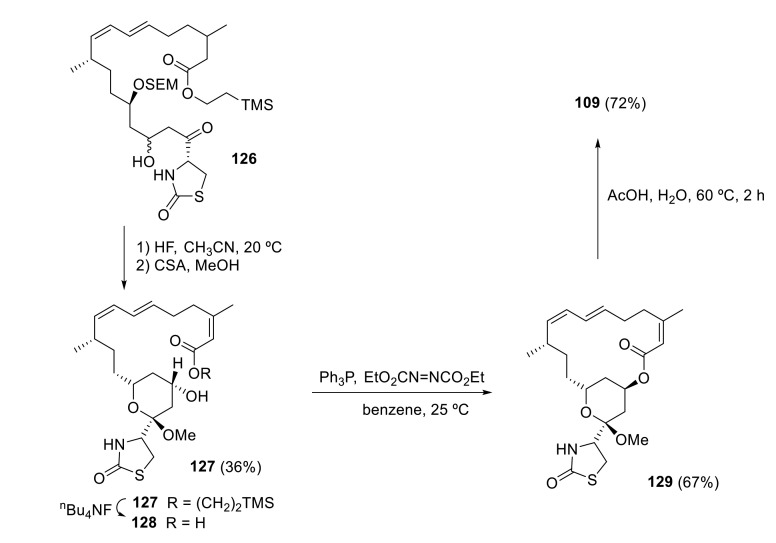
White’s synthesis of latrunculin A.

Whites’ synthetic approach for latrunculin A **109** goes via the advanced intermediate **126** [94]. After selective deprotection of the SEM ether with concentrated HF, the tetrahydropyran ring **127** is constructed by exposing the resulting diol to acidic (CSA) methanol. Then, ester cleavage to **128**, followed by Mitsunobu reaction, gives macrolactone **129**. Simple hydrolysis with acetic acid affords the desired latrunculin A **109** (Scheme 35).

## 4. Heterocyclic Marine Drugs with Effect on Tumor Cell Proliferation

### 4.1. Mandelalides A–D

Mandelalides A–D are several glycosylated macrolides isolated from a new species of *Lissoclinum ascidian* that can be found in South Africa, Algoa Bay, close to Port Elizabeth and the surrounding Nelson Mandela Metropole [95]. These compounds show strong cytotoxicity to both human and mouse cancer cells, including neuroblastoma and lung cancer cells. The action mechanism is related to the inhibition of mitochondria complex V in the nM range. Specifically, treatment with mandelalide A decreases the aerobic respiratory capacity of living cancer cells dramatically [96]. By disrupting the mitochondrial membrane potential, mandelalide A destroys the main driving force for the mitochondrial Ca^2+^ uptake indirectly. If calcium cannot enter mitochondria, the store-operated calcium entry is inhibited, which is the main Ca^2+^ entry pathway involved in many tumor cell proliferation. Thus, it may lead to tumor cell cytotoxicity [97].

**Figure 11:**
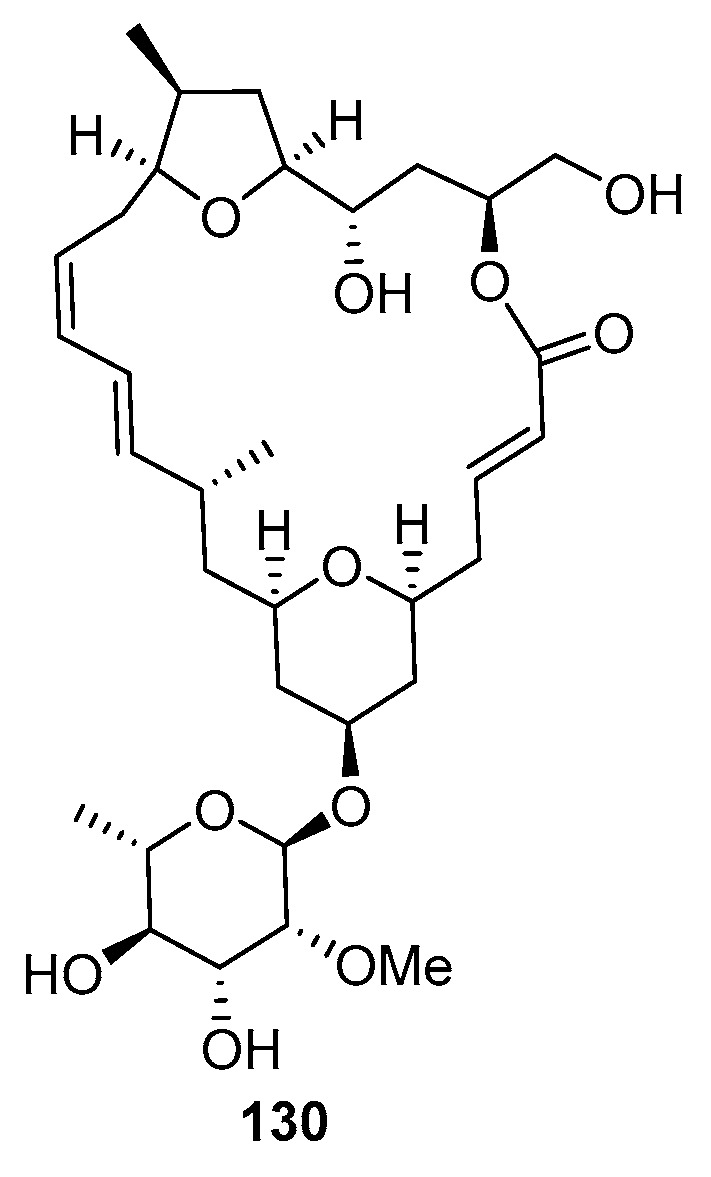
Structure of mandelalide A (**130**).

Two bicyclic domains are part of the overall macrocyclic macrolides, a (glycosylated) 2,6-*cis*-substituted tetrahydropyran and 2,5-*cis*-substituted tetrahydrofuran (Figure 11). Altmann has described a highly efficient convergent total synthesis of madelalide A, in which different methodologies have been used for the construction of the two main heterocycles of this macrolide.

**Scheme 36:**
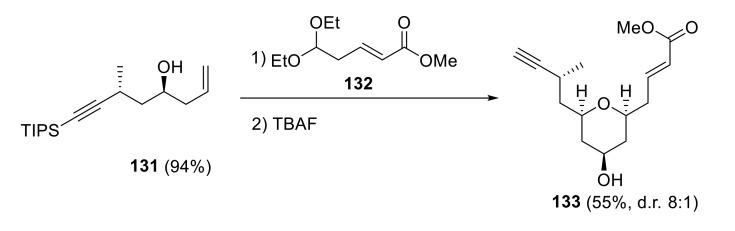
Synthesis of the tetrahydropyran unit.

**Scheme 37:**
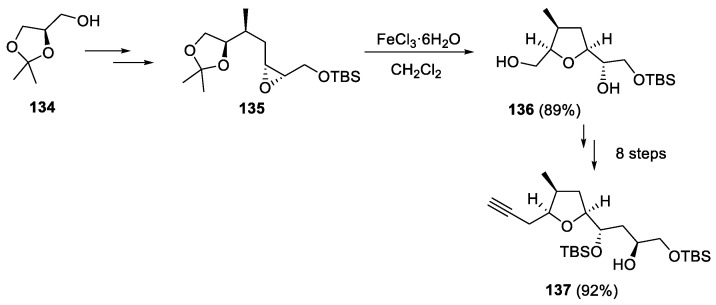
Synthesis of the tetrahydrofuran building block **137**.

Thus, the tetrahydropyran unit of building block **133** was prepared by a Prins cyclization reaction of the homoallylic alcohol **131** and the acetal **132** (Scheme 36). On the other hand, the synthesis of tetrahydrofuran moiety **137** included more steps and departed from the commercially available acetonide **134**. The five-membered oxacycle **136** was obtained by reaction of the intermediate **135** with FeCl_3_·6H_2_O, in a tandem reaction involving acetonide cleavage and the subsequent attack of the liberated secondary hydroxy group to the proximal epoxide carbon (Scheme 37).

### 4.2. Patellamide

Patellamide is a cycle peptide and natural product isolated from *Prochloron didemni*, a cyanobacterial symbiont of *Lissoclinum patella*. This compound also shows strong cytotoxic and growth inhibitory activity in cancer cells [98]. The mechanism by which patellamide inhibits tumor cell proliferation is unknown, but its effects have been compared to verapamil, a well-known Ca^2+^ channel blocker also cytotoxic in cancer cells. Calcium entry is mandatory for cell proliferation; any compound that inhibited calcium entry in a sustained way could inhibit cell proliferation [99].

**Figure 12:**
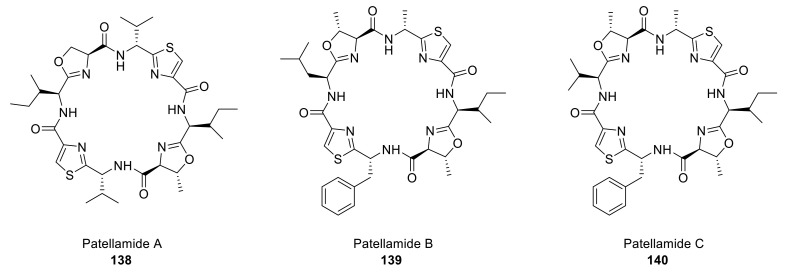
Structures of the patellamide family.

Patellamides A–C contains two oxazoline and two thiazole subunits in their structure (Figure 12).

**Scheme 38:**
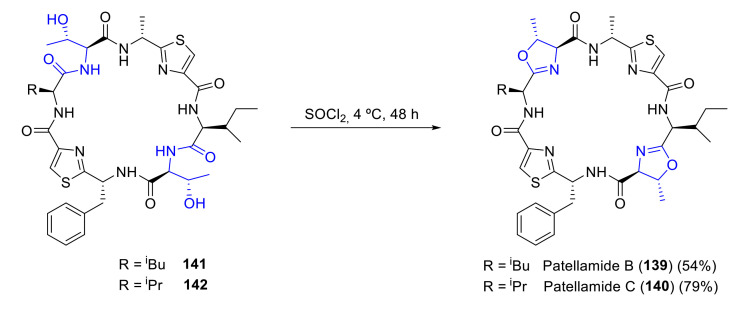
Synthesis of patellamides B and C by Hamada and Shioiri.

The original synthetic methodology for patellamide B and C proposed by Hamada and Shioiri is based on the construction of the cyclic peptide structure from peptide building blocks and late formation of the oxazoline ring [98]. All peptides used in this route are previously synthesized from thiazole amino acids following a developed procedure [100]. Patellamides B and C are analogously synthesized but with different initial amino acids. In the last step, intermediates **141** and **142** are treated with SOCl_2,_ thus forming the oxazoline rings and yielding the desired compounds (Scheme 38). A similar methodology was used to prepare patellamide B [101].

**Scheme 39:**
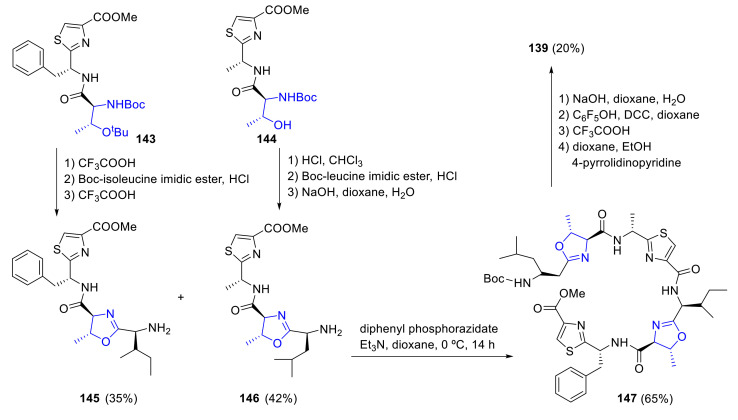
Schmidt’s synthesis of patellamide B.

Schmidt proposed a different approach to patellamide B [102]. In this route, the oxazoline rings are formed early in the synthesis by reacting protected aminoalcohols **143** and **144** with boc-isoleucine imidic ester. Coupling of the two fragments **145** and **146** with diphenyl phosphorazidate produced a linear peptide **147**, which cyclization then affords patellamide B **139** (Scheme 39).

**Scheme 40:**
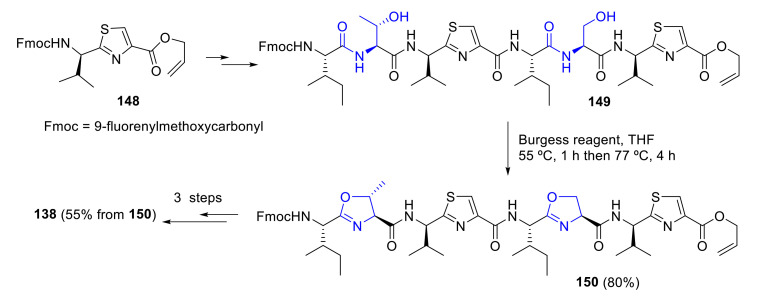
Synthesis of patellamide A by VanNieuwenhze.

More recently, a new approach relying on contemporary methods for the construction of the oxazoline rings was proposed by VanNieuwenhze [103]. In this strategy, all thiazole and oxazoline rings are installed in a linear intermediate, leaving the macrocyclization for the last step. Starting from thiazole **148**, intermediate **149** was synthesized following a convergent condensation of peptide fragments. By treatment with Burgess reagent, both oxazoline subunits are formed through a cyclodehydration process yielding compound **150**. Then, cleavage of both the allyl ester (PdPPh_3_)_4_ and the Fmoc group (piperidine/DMF) and macrocyclization (PyBOP, DMAP, DIEA) yielded the desired patellamide A **138** (Scheme 40).

### 4.3. Lamellarins

Lamellarins are natural alkaloids originally isolated from a small sea snail that show potential antiviral and anticancer properties. More than 50 lamellarins are available at present, being lamellarin D the leading compound of most of them. Lamellarins are frequently used as starting points for the design of anticancer drugs [104]. Lamellarin D shows strong inhibitory capacity of both nuclear and mitochondrial topoisomerase I, a protein involved in DNA reparation. It also interacts directly with mitochondria to elicit the death of cancer cells. In fact, the antitumoral effect of lamellarin D is seemingly mediated by apoptosis activation even in multidrug-resistant cancer cell lines. Lamellarin D acts as a direct inductor of mitochondrial permeability transition pore. Treatment with lamellarin D significantly reduces the threshold level of calcium required for opening the mitochondrial permeability transition pore induction. This leads, accordingly, to caspase-independent mitochondrial depolarization, mitochondrial swelling and release of cytochrome c. All these data indicate that lamellarin D facilitates calcium-dependent mitochondrial permeability transition pore opening leading to apoptosis in cancer cells [105].

**Figure 13:**
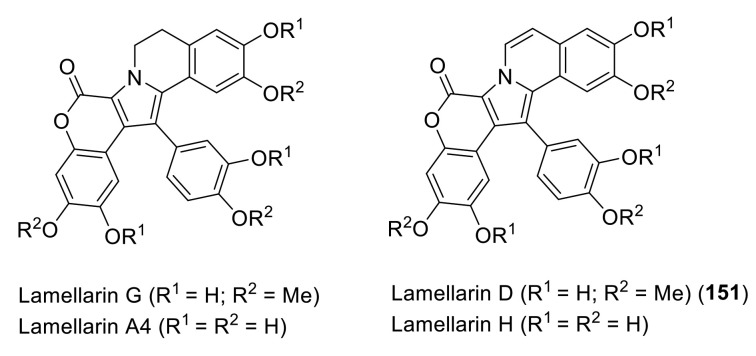
Structures of some members of the lamellarin family.

Lamellarins A–D were isolated in 1985 by Faulkner and coworkers from the marine mollusk *Lamellaria* sp. [106]. Since then, more than 70 lamellarins and related compounds have been isolated from mollusks, sponges, and tunicates. The structures of some members of the family are depicted in Figure 13, although we will only focus on lamellarin D. Two approaches are possible to build the central functionalized pyrrole ring. The first one includes the functionalization of the pyrrole moiety, and the second one consists of employing the appropriate acyclic precursor to synthesize the pyrrole. Due to its biological activity, many groups have devoted their time to develop methods for its synthesis [107,108,109,110] as well as the synthesis of other members of the family [111,112,113,114].

**Scheme 41:**
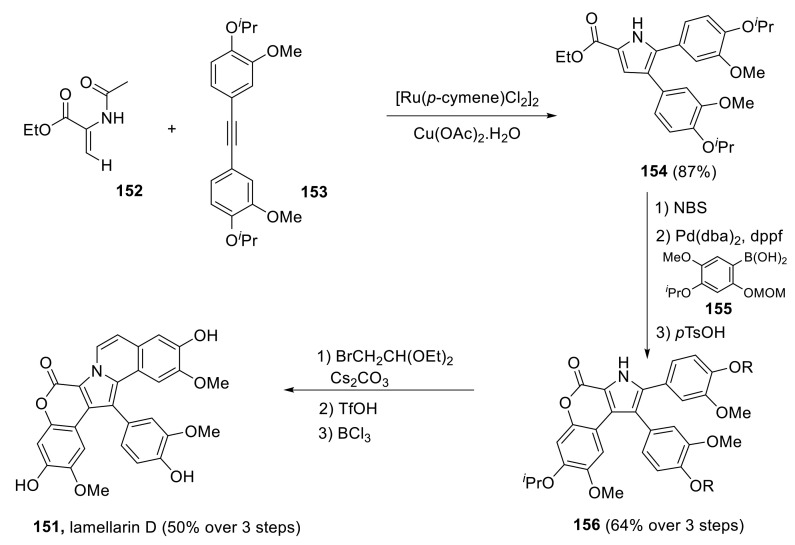
Total synthesis of lamellarin D by Chandrasekhar.

In 2017, Chandrasekhar et al. proposed a total synthesis of lamellarin D in which the central pyrrole ring was synthesized through Ru(II)-catalyzed (3 + 2) annulation strategy [115]. The total synthesis of lamellarin D **151** was carried out in seven steps and in a 29% overall yield. The key intermediate **154** was obtained via Ru-catalyzed oxidative annulation of enamide **152** and diarylalkyne **153** in 87% yield. Bromination of this product, followed by Suzuki–cross-coupling with **155** and final MOM deprotection/lactonization, provided tetrasubstituted pyrrole **156** in 64% yield over three steps. N-alkylation and intramolecular Iwao cyclization generated an intermediate, which was treated with BCl_3_ to furnish lamellarin D **151** (Scheme 41).

**Scheme 42:**
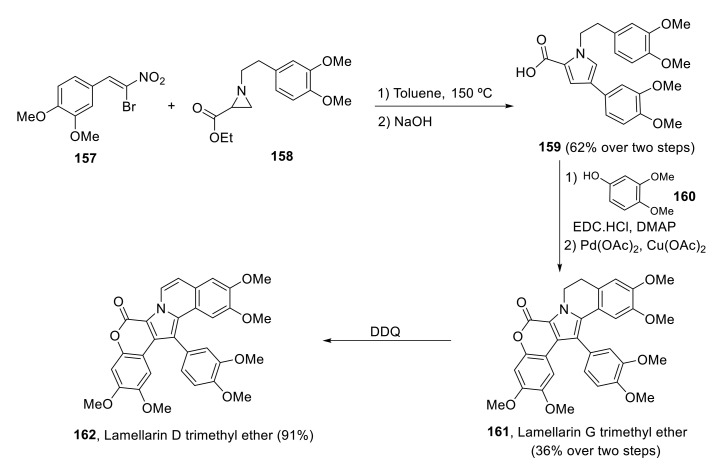
Khan’s synthesis of lamellarin D trimethyl ether.

Khan et al. carried out a short and scalable total synthesis of lamellarin D trimethyl ether **162** and other analogs in 2019 [116]. The construction of the pyrrole ring was performed by annulation of aziridine ester **158** with β-bromo-β-nitrostyrene **157,** giving the central 1,2,4-trisubstituted pyrrole core in a highly regioselective manner. Coupling of pyrrole-2-carboxylic acid **159** with phenol **160**, followed by Pd-catalyzed intramolecular oxidative C-H arylation, afforded the pentacyclic **161**, lamellarin G trimethyl ether, in 44% yield. Lamellarin D trimethyl ether **162** was obtained by dehydrogenation with DDQ in excellent yield (Scheme 42).

**Scheme 43:**
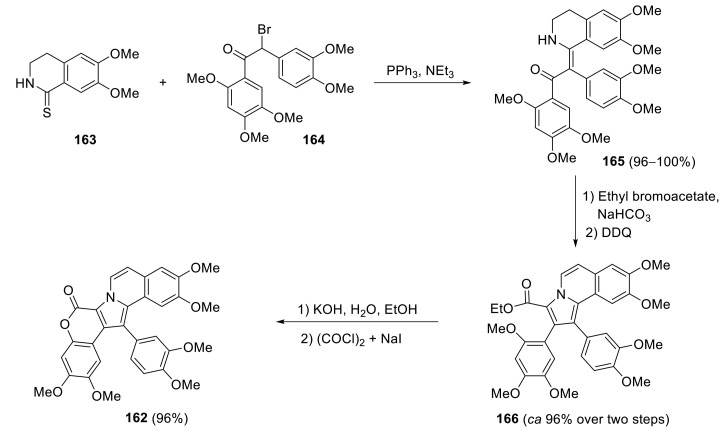
Michael’s synthesis of lamellarin D trimethyl ether.

More recently, Michael and coworkers have published the synthesis of lamellarin D trimethyl ether **162** by using enaminones as intermediates to alkaloids and other nitrogen heterocycles [117]. Eschenmoser sulfide contraction between bromoketone **164** and thiolactam **163**, in the presence of triphenylphosphine and trimethylamine, afforded the desired heptamethoxylated enaminone **165** in high yields (96–100%). Conventional heating of enaminone **165** in ethyl bromoacetate provided a tricyclic product, which was oxidated by DDQ to give the unsaturated ring **166**. A final saponification/lactonization process furnished lamellarin D trimethyl ether **162** in yields consistently above 96% (Scheme 43). Overall, yield over seven steps was 86%, the highest reported to date.

## 5. Conclusions

In this review, we have tried to show an overview of a group of marine drugs that, from the structural point of view, contain one or various heterocyclic units in their core structure and, from the biological side, have a direct influence on the transport of calcium in the cell. The marine compounds covered in this review are divided into three groups, which correspond to their direct biological activity, such as compounds with a direct influence in the calcium channel, compounds with a direct effect on the cytoskeleton and drugs with an effect on cancer cell proliferation. For each target, we have described its bioactive properties and synthetic approaches. The wide variety of chemical structures compiled in this review and their significant medical properties will attract the attention of many researchers.

## Data Availability

Not applicable.
